# Toward Smart Implant Synthesis: Bonding Bioceramics of Different Resorbability to Match Bone Growth Rates

**DOI:** 10.1038/srep10677

**Published:** 2015-06-02

**Authors:** Rafael Comesaña, Fernando Lusquiños, Jesús del Val, Félix Quintero, Antonio Riveiro, Mohamed Boutinguiza, Julian R. Jones, Robert G. Hill, Juan Pou

**Affiliations:** 1 Applied Physics Dpt., University of Vigo, E.I.I., Lagoas-Marcosende E-36310, Vigo, Spain; 2 Department of Materials, Imperial College London, South Kensington Campus, London SW7 2AZ, United Kingdom; 3 Unit of Dental and Physical Sciences, Barts and the London, Mile End Road, London E1 4NS, United Kingdom

## Abstract

Craniofacial reconstructive surgery requires a bioactive bone implant capable to provide a gradual resorbability and to adjust to the kinetics of new bone formation during healing. Biomaterials made of calcium phosphate or bioactive glasses are currently available, mainly as bone defect fillers, but it is still required a versatile processing technique to fabricate composition-gradient bioceramics for application as controlled resorption implants. Here it is reported the application of rapid prototyping based on laser cladding to produce three-dimensional bioceramic implants comprising of a calcium phosphate inner core, with moderate *in vitro* degradation at physiological pH, surrounded by a bioactive glass outer layer of higher degradability. Each component of the implant is validated in terms of chemical and physical properties, and absence of toxicity. Pre–osteoblastic cell adhesion and proliferation assays reveal the adherence and growth of new bone cells on the material. This technique affords implants with gradual-resorbability for restoration of low-load-bearing bone.

The bone tissue regeneration approach involves placing a temporary template into a bone defect that will promote cell attachment, proliferation, differentiation, three-dimensional vascularised bone growth, with minimal fibrous tissue growth followed by degradation as the bone remodels. This approach is necessary in the repair of defects due to traumatic accidents, tumour extraction surgery, or congenital deformities correction. The autograft utilization has potential complications and it is not always available in sufficient quantity^1^,^2^,^3^; therefore, synthetic biomaterials are employed for such applications. A further development is the use of implants that are tailored to the patient, as schematized in Fig. 1a. Critical size defects are not expected to grow back to normal size and shape by themselves, hence they required aid of a bone substitution material. The new bone ingrowth rate depends on several factors, such as the defect location, the surrounding bone type, the graft material, and the patient age and health. Nevertheless, the tracking studies of the healing process reveal a common pattern, showing the most intensive osteogenesis within an initial stage, and followed by a remarkable reduction of bone ingrowth rate at medium and long-term periods^4^,^5^,^6^. Patients would benefit from tailored bioactive implants with resorbability capable to match the new bone growth rate at different healing stages^7^,^8^,^9^,^10^,^11^. Gradual resorption is the main objective in order to provide high resorbability and osteoinduction in the first stage after implantation, in addition to long-term implant stability^8^,^9^,^11^.

Most calcium phosphate (CaP) bioceramics show very good biocompatibility as well as osteoconductive and osteoinductive capabilities^12^,^13^,^14^. The ions released during physiological fluid and osteoclast mediated dissolution stimulate the expression of different genes related to bone production^15^,^16^,^17^,^18^,^19^, but the resorbability frequently differs from the bone ingrowth kinetics^20^,^21^,^22^,^23^. With the aim of increasing resorption rates, the development of doped CaPs and the combination of CaP compounds with bioactive glasses (BGs) have been assessed^24^,^25^,^26^. The silica based BGs are osteoconductive and their dissolution products up-regulate several important genes related to bone formation^27^,^28^,^29^,^30^,^31^. As a result, the effect of the BG resorption mediated by the physiological fluids mimics those bone matrix-derived factors released during osteoclastic bone resorption in biological remodelling^32^,^33^,^34^, increasing the preosteoblastic cells differentiation and production of new bone. Nevertheless, the *in vivo* degradation of BGs is too fast to allow the required stability for new bone formation at long-term^35^,^36^,^37^. Processing homogeneous CaP and BG mixtures affords ceramics and glass–ceramics composites that exhibit intermediate but, nevertheless, spatially homogeneous *in vivo* degradation^38^,^39^,^40^. The approach to overcome this limitation is to selectively modify the bioceramic in the spatial domain.

In this study, we present a new laser-assisted fabrication method for customized bone implants designed for patients undergoing reconstructive surgery. The unique implant is composed of a CaP modified inner core with moderate degradation rate, and a BG outer layer of higher degradability, as explained in Fig. 1b. This technique provides a method to produce gradual resorbability implants for low load bearing bone restoration, with capabilities to integrate specific agents inducing antibacterial, angiogenic, or antiresorptive activity. Laser cladding has already been applied in its bi-dimensional version to produce calcium phosphate coatings^41^,^42^,^43^,^44^,^45^,^46^ and bioactive glass coatings^47^ on titanium alloys for biomedical applications.

## Results

**Figure 2:**
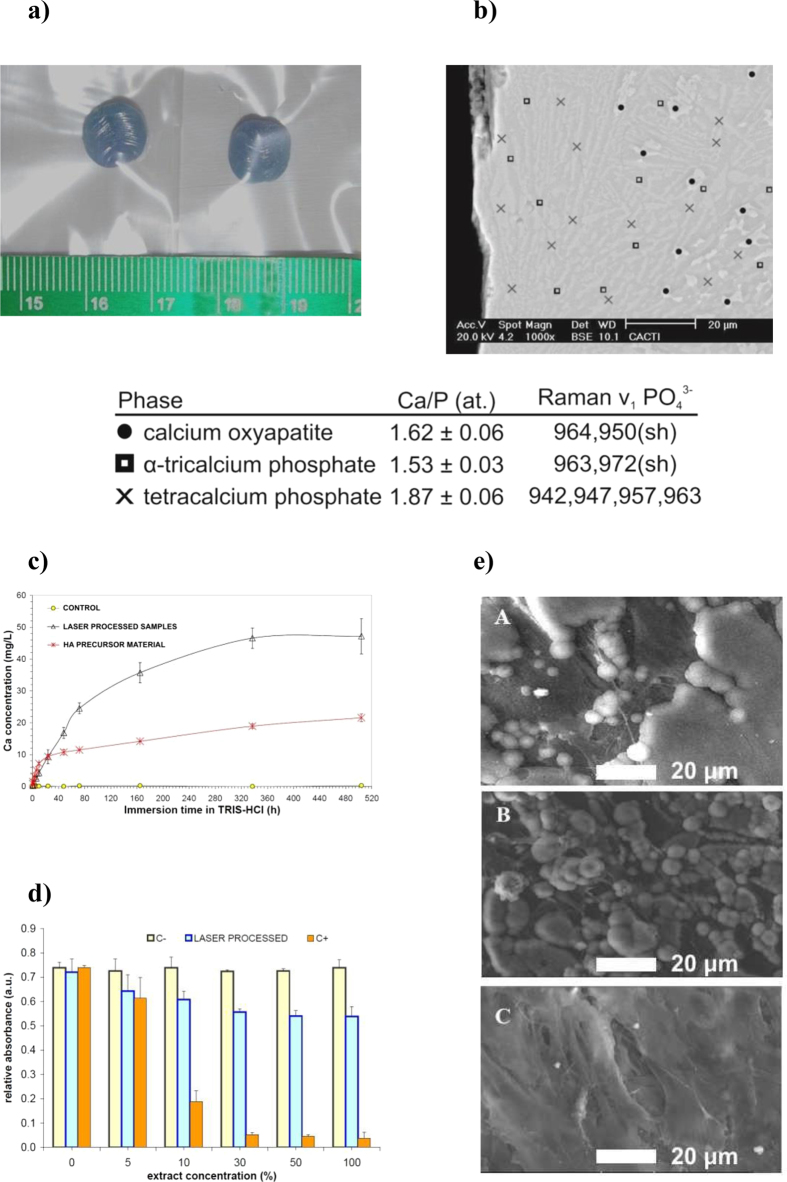
Synthesis and validation of inner core implant material by laser cladding of HA. (**a**) CaP samples produced by rapid prototyping based on laser cladding (laser wavelength: 1064 nm, 50 stacked layers). (**b**) Backscattered electron micrograph of the cross section near the surface showing metastable α-TCP matrix surrounding elongated TTCP grains and internal oxyapatite grains. (**c**) Calcium ion release during dissolution in Tris-HCl buffer. (**d**) MC3T3-E1 proliferation in different dilutions of laser-cladding-processed HA extracts (statistical significance p < 0.05; C- pure cell culture medium). (**e**) SEM images of attachment and spreading of the MC3T3-E1 cell line on the laser-cladding-processed sample surfaces (A 1 day, B 3 days, C 7 days).

The low resorption inner core of the implant was synthesized using highly crystalline stochiometric HA as bioceramic precursor. In order to increase the material reactivity, the precursor particles were subjected to powerful laser [wavelength 1064 nm] irradiation sufficient to induce the following reactions: (1) debonding of solid state hydroxyl groups from the HA lattice (Ca_10_(PO_4_)_6_(OH)_2_) leading to the formation of oxyapatite (Ca_10_(PO_4_)_6_O_2_); and (2) partial decomposition of oxyapatite and phase change to a liquid phase in the CaO–P_2_O_5_ system. The subsequent step in the process involves displacement of the deposited material away from the laser irradiated area and cooling at moderate rates. This step leads to the precipitation from the liquid phase of tetracalcium phosphate (TTCP, Ca_4_(PO_4_)_2_O) and alpha-tricalcium phosphate (α-TCP, α-Ca_3_(PO_4_)_2_). As a result, the obtained implant presents a unique multiphasic microstructure, which keeps the overall material Ca/P ratio and combines highly resorbable CaP phases with still-lower-resorbability oxyapatite (Fig. 2a and b). Complete dehydroxylation of the HA particles during processing of multilayered samples is demonstrated by Raman and Fourier-transform infrared (FTIR) spectroscopy (see supporting information). The flow of injected particles, the absorbed energy density, and the coaxial Argon flow govern the thermal cycle experienced by the HA and, therefore, the proportion of molten oxyapatite preceding the formation of α-TCP and TTCP phases. The identification of each crystalline phase was performed by micro-Raman and quantitative energy dispersive X-ray spectroscopy using CaP standard materials with certified Ca/P ratios (table in Fig. 2).

The generation of an intermediate liquid phase is a key feature of this laser-assisted technique that is necessary to obtain fine homogeneous layers. It avoids the requirement of additional binding substances (which are toxic or reduce bioactivity) to consolidate the CaP according the geometry defined in the preoperative evaluation and surgical planning. In addition, the presence of a liquid phase with lower surface tension leads to oxyapatite-free surfaces, with implications for implant resorbability and bioactive behavior, and particularly for the possibility of chemical reactions during further processing with dissimilar bioceramic materials such as silicate BGs. Macroscopic CaP samples are produced by stacking a variable number of layers: typically, samples are processed from 20 to 320 stacked layers with layer thicknesses between 100 to 300 micrometers and a 100 °C/min cooling rate to preserve the material integrity (see Fig. 2a).

To demonstrate the increased degradability in comparison with stoichiometric HA, the ion release profiles of the fabricated implants in physiological conditions were tested by immersion in 0.05 M Tris–HCl buffer. Figure 2c shows that the calcium concentration released by the laser processed material is remarkably higher than that released by the precursor HA. The TTCP and metastable α-TCP generated by this process present a lower chemical stability under physiological pH than stoichiometric calcium HA. Additionally, we demonstrated the non-cytotoxic behavior of the CaP implant cores by means of a solvent extraction test and seeding of the pre-osteoblastic line MC3T3-E1 (Fig. 2d). The capacity of bone cell precursors to adhere to the inner core during the healing process was assessed by scanning electron microscopy. Figure 2e shows that the cells seeded on the culture plates adhered to the material and proliferated over time to cover the surface completely after 7 days. These experiments demonstrate the low toxicity of the material, and agree with previous findings stating that TTCP and α-TCP phases are not detrimental to proliferation of pre-osteoblastic cells and promote cell differentiation and extra-cellular matrix mineralization^15^. Considering the existing calcium phosphate phases in the implants, it can be expected that the implants allow bone cells to develop at their surface during the healing process. Another important feature of the laser-engineered implants is that the Ca/P ratio in the samples at the few-micrometer scale remains the same as that in the precursor material, which prevents the excessive occurrence of harmful intermediate reactions during dissolution and biological apatite precipitation, and limits the pH induced change in the environment.

### Outer Shell: Laser Cladding of Highly Resorbable Bioactive Glass

In order to produce higher-resorbability outer layers, we first investigated the applicability of silicate-based BG microparticles to the process. The precursor particles were injected into the molten pool at high speed, where the absorbed laser radiation increased the temperature and melted the particles in several milliseconds. We started the process over a Ti6Al4V disposable substrate and it was afterwards self-supported due to the immediate increase in glass viscosity as the temperature decreased. As described above for the preparation of the CaP inner core, the relative movement of the injecting nozzle and laser beam with respect to the substrate is controlled to deposit the glass in a layer-by-layer manner. The processing parameters are adjusted to maintain the temperature of the deposited BG above the transition temperature for long enough to avoid thermal shock. In selecting the precursor glass composition we considered the reported biocompatibility, osteoconductivity, and osteoinductivity in addition to the devitrification tendency and viscosity-temperature evolution^7^,^26^,^27^,^28^. The 45S5 Bioglass® composition developed by Hench is considered a reference in terms of bioactivity. However, this material is prone to devitrification during hot working processes^48^,^49^ and only very fast processes such as laser spinning are able to preserve the amorphous state^50^. Devitrification can cause instability during dissolution and compromises the mechanical consistency of the implant material due to tensions caused by the density mismatch between the grown crystallites and the parent glass. This leads to an increase in eventual failure probability and even complicates implant handling. Therefore, we employed an alternative BG composition, the S520 BG, to avoid the above mentioned problem. Crystal growth inhibition by potassium addition allows S520 BG to have a smooth viscosity-temperature evolution and a higher working range, combined with the required bioactivity^47^,^51^. Thus, we were able to increase the processing window and the implant size while maintaining the desired solidity.

**Figure 3:**
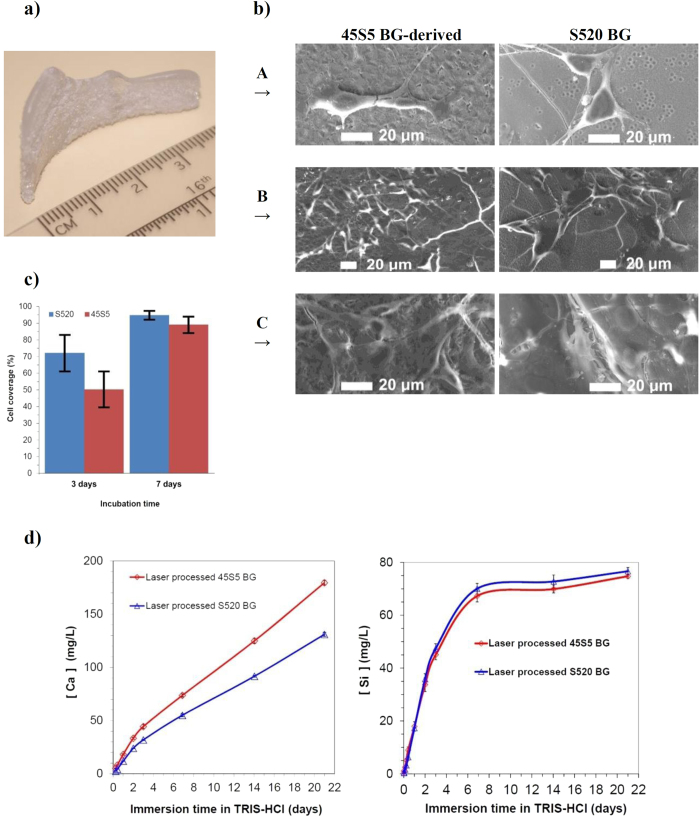
Validation of the outer shell material. (**a**) Layered bioceramic implant produced by rapid prototyping based on laser cladding of S520 BG from a three-dimensional CAD model of a left orbital rim implant. **b**) SEM micrographs showing the attachment and spreading of MC3T3-E1 cell line (A 1 day, B 3 days, C 7 days). (**c**) Cell coverage of the sample surface after 3 and 7 days of incubation time (statistical significance *p* < 0.05). (**d**) Ion concentration after different times of immersion in 0.05 M Tris-HCl buffer for laser engineered 45S5 and S520 BG samples.

The properties of the outer shell material were validated by scanning electron microscopy, cell proliferation assay, and immersion test. Figure 3a shows a layered bioceramic implant produced by irradiation of exclusively S520 BG. Nucleation is present via a surface crystallization mechanism, but the crystal growth is strongly inhibited, barely reaching a depth of a few microns under the implant surface and not compromising the implant function. Comparison of precursor material and processed samples by X-ray fluorescence analysis showed differences that were within the intrinsic compositional variability of the precursor glass, which supports the preservation of the precursor glass chemical composition. In addition, we demonstrated the non-cytotoxic behavior of the laser-processed BG by means of a solvent extraction test and seeding of the pre-osteoblastic line MC3T3-E1. Comparative cell proliferation assays showed very similar results for laser processed S520 and 45S5 BGs^52^. The assessment by means of scanning electron microscopy showed the ability of bone precursor cells to adhere to the BG surfaces. In the on-sample direct seeding tests, the pre-osteoblastic cells that attached to the implant surface exhibited a healthy morphology and spread over the complete sample surface after seven days of incubation (Fig. 3b). Measurements of the osteoblast coverage of surface area show that approximately 60% and 90% of surface is covered after 3 and 7 days of incubation time, respectively. The statistical analysis reveals that differences of coverage between the samples obtained from 45S5 and S520 BGs are not significant (statistical significance *p* < 0.05).

The ion release profile of calcium and soluble silica from BGs leads to the up-regulation and activation of different families of genes in osteoprogenitor cells with important consequences in rapid bone regeneration^29^,^30^. Particular concentrations of these ions, 60-80 g/mL and 16-50 g/mL of calcium and soluble silica, respectively, promote proliferation and differentiation of the mature osteoblast phenotypes, such as cell cycle regulators, apoptosis regulators, synthesis of DNA or growth factors^28^,^53^. The dissolution rates and apatite precipitation of these laser-engineered implants in physiological fluids remains analogous to that of the precursor glass, as shown in Fig. 3d. From these results, it is possible to predict the implant dissolution rate as a function of the precursor composition and thus to design and fabricate BG implants with tailored resorbability in the spatial domain. Nevertheless, the high silica content required to achieve low resorption would lead to loss of the osteoconduction^35^. Therefore, bioactive implants made of BG particles would be more practical if combined with a dissimilar low-resorbability bioactive material. Thus, we present subsequently the application of this laser-processed BG as a covering layer for a CaP core implant to obtain a material showing gradual resorbability.

### Inner Core-Outer Shell Coupling into a Gradually Resorbable Material

**Figure 4:**
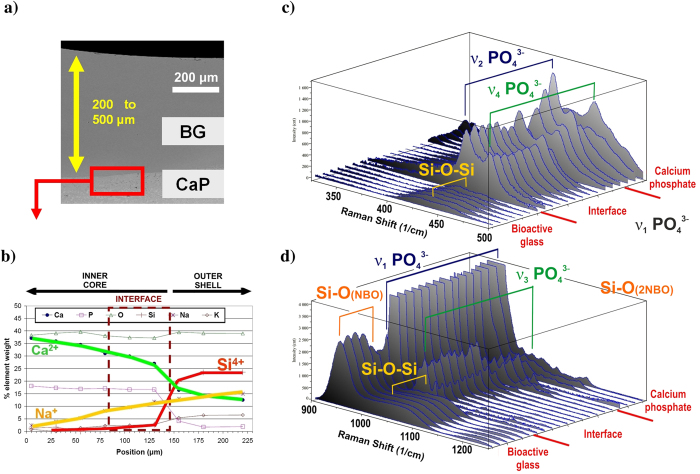
Elemental composition and linear scanning of Raman shift spectra at the interface between the CaP core and the S520 BG outer layer. (**a**) Cross-sectional SEM overview of the outer S520 BG cover. (**b**) Elemental composition obtained by energy dispersive X-ray spectroscopy (EDS) microanalysis across the interface zone. (**c**) and (**d**) Raman spectra across the interface zone.

In order to create a transition between the highly resorbable BG surface and the CaP multiphasic core, we deposited BG layers on previously processed CaP implants. Once the inner core is completed by adding the last CaP layer, and the temperature of the material has decreased to 500 °C during the cooling process, the BG particles are sprayed on its surface using a secondary nozzle and laser irradiation is applied. Essentially, the consolidated CaP inner core becomes a substrate for the outer BG layers. Glass thicknesses from 200 μm to 500 μm can be deposited in a single layer (Fig. 4a), but higher thicknesses can be achieved by stacking successive layers in a direction perpendicular to the surface.

**Figure 5:**
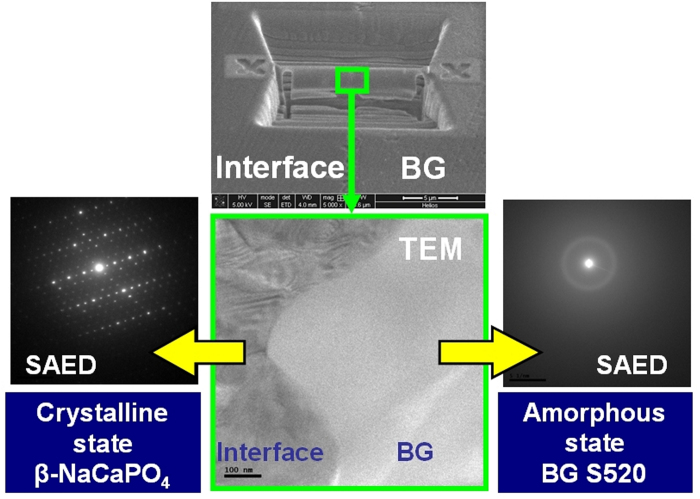
TEM analysis of the interface between the CaP inner core and outer BG layer. (Upper center) SEM micrograph showing the area removed by FIB technique and the remaining lamella between the interface and the deposited glass. (Lower center) Transmission electron micrograph of the lamellae center showing the crystalline-amorphous border. (Lower left) The SAED of the interface revealing the characteristic diffraction pattern of β-rhenanite (β-NaCaPO_4_). (Lower right) The SAED of the very near BG to the interface confirms the amorphous state.

**Figure 6:**
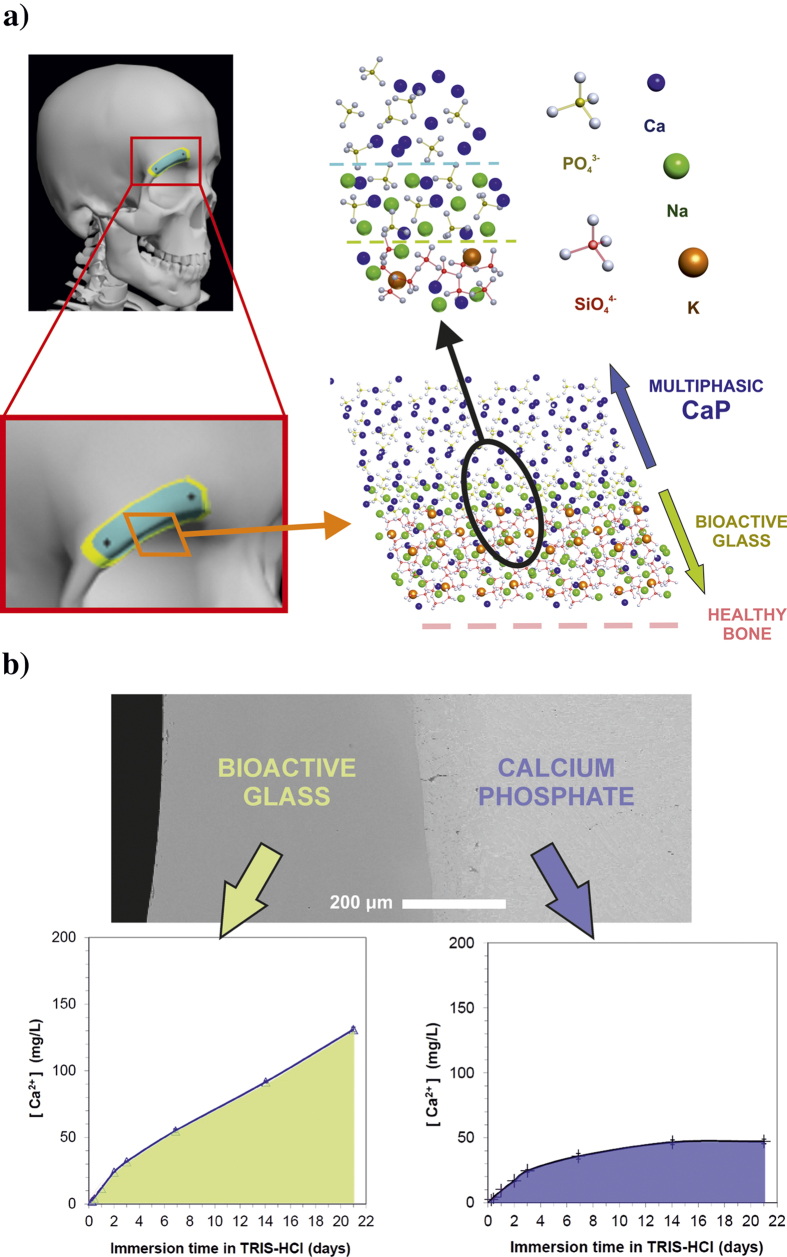
Schematic illustration of the structural arrangement in the patient-tailored bioceramic implant, and calcium release profile of surface and inner core. (**a**) The implant surface just neighboring the trepanned bone edge comprises a disordered silicate tetrahedral network, opened by an elevated concentration of modifier cations. The highly reactive BG structure is smoothly connected to the less-reactive multiphasic monoclinic arrangement of phosphate tetrahedrons and Ca^2+^ cations through an orthorhombic crystal lattice with decreasing presence of Na^+^ cations. (**b**) Ca^2+^ release profile in Tris-HCl buffer for HA precursor material, bare multiphasic CaP before laser cladding with BG, and of samples with S520 BG outer layers.

Insofar as this region will be subjected to degradation by cells and physiological fluids, structural modifications and new chemical compounds formed at the interface can compromise the bioactivity of the implant. At the interface between CaP and the BG (see Fig. 4b), cation diffusion occurs to afford a chemically homogeneous band by the solid-state reaction of sodium and CaP. Calcium ions are released from the interface and sodium calcium phosphate (β-rhenanite) is formed; the presence of this compound was demonstrated by micro-Raman (Fig. 4c and d) and FTIR spectroscopy (supplementary figure S1). The Raman and FTIR spectra obtained from the interface are in good agreement with the reported spectra for β-NaCaPO_4_. Lamellae were extracted using the focused ion beam (FIB) technique for transmission electron microscopy analysis of the interface, as shown in Fig. 5. The selected area electron diffraction (SAED) patterns demonstrated the amorphous state of the glass just beside the interface and the crystallinity of the interface. The obtained inter-planar distances confirm the presence of interfacial β-rhenanite. The coupling of amorphous and crystalline structural arrangements within the processed bioceramic implants is schematically represented in Fig. 6a. During a potential surgical implantation, the implant would be placed after resection of the damaged bone. To optimize the implant response, highly reactive BG must be immediately accessible by the healthy tissue of the implant site boundaries. On the contrary, the CaP material located far from the boundaries has to enable the new bone ingrowth without losing the implant stability. The control of the BG layer thickness is required to fulfill this function, in addition to avoid formation of harmful or less bioactive compounds at the interface with the CaP core. In this work, the connection between the highly reactive silicate based glass and a more stable phosphate monoclinic structure is provided through the orthorhombic crystal lattice of a gradually substituted β-rhenanite, formed by the solid-state reaction between α-TCP, TTCP, and Na^+^ from the coating.

The interconnecting β-rhenanite phase is a biocompatible and resorbable material that stimulates bone growth. The expression of cellular proliferation osteogenic markers in the presence of β-rhenanite was found to be higher than that in the presence of β-tricalcium phosphate. Granular bone substitutes containing β-rhenanite are approved as bone defect fillers for clinical use and are available on the market^54^,^55^,^56^. On the contrary, while apatite formation in simulated body fluid is reported for other intermediate compounds formed by thermal processing of silica glass and CaP, such as Ca_5_(PO_4_)_2_SiO_4_ and Na_3_Ca_6_(PO_4_)_5_, no experimental evidence of biocompatibility has been demonstrated through cytotoxicity assessments. With our technique, the formation of such additional phosphate phases at the interface is prevented by the absence of oxyapatite on the surface of the CaP core (see Fig. 2a). In the laser-processed implants presented here, the likelihood of silicate ion diffusion beyond the interface is minimal. The laser power density and associated sodium diffusion are limited to avoid the possibility of Na_3_Ca_6_(PO_4_)_5_ formation by a reaction of the oxyapatite grains present underneath the CaP core surface. Therefore, a suitable biological response is to be expected; the β-rhenanite compound formed at the interface between the CaP core and the BG outer layer does not compromise the bioactivity of the laser-processed implant.

The structural changes of the BG outer shell were measured after the process by sequential micro-Raman spectra acquisition at different distances from the interface. The network connectivity is strongly related to the glass bioactivity^57^, glasses with high network connectivity (greater than 2.4) are characterized by low degradability and an inert biological response, while highly disrupted networks result in faster dissolution. We observed that the glass structure is remarkably more open in regions close to the interface zone, which means that no passivation layer is produced at this point. The glass structure closes from the interface outwards, reaching the precursor glass structure connectivity at a few micrometers from the interface. Due to the low crystallization tendency of the S520 BG, from this point near the interface to the surface, the Raman spectra are identical to that of the precursor material and, therefore, the glass structure and its reactivity is consistent with the employed precursor glass.

The mechanical properties of these laser engineered implants enable their use in low-load-bearing applications. The compressive strength, 359 ± 51 MPa, is well above the cortical bone compressive strength^35^. The hardness of the bioactive glass outer layer and CaP core, 454 ± 30 and 443 ± 19 HV, and the fracture toughness *K*_IC_ 0.91 ± 0.13 and 1.29 ± 0.17 MPa·m^1/2^, respectively, match the reported values for bioactive glasses and CaP sound ceramics^35^,^58^.

Owing to the sequential processing of CaP and BG layers by rapid prototyping based on laser cladding, we obtained unique implant materials comprising a multiphasic CaP core surrounded by a BG covering. According to the degradability tests in physiological media, the BG external layers are expected to present high *in vivo* reactivity, adequate to the bone growth rates at early implantation times, while the multiphasic CaP core provides lower degradability rates and guarantees implant function in the long term (see Fig. 6b). The amount of each material in the implant can be tailored, thus allowing adaptation of the resorption and osteoconduction levels at each location within the implant and improving its gradual substitution by newly formed bone. Moreover, the particle injection characteristics allows enhancement of the surface with specific features of biological relevance, for instance antibacterial or angiogenic activity, by means of incorporation of additional particles such as Ag_2_O, ZnO, Co_3_O_4_, or SrO.

## Conclusion

We demonstrated the feasibility of producing unique implants for low-load-bearing bone repair through the application of rapid prototyping based on laser cladding using CaP and BG particles. The processing method is characterized by the use of a high power laser beam for the production of a self-sustained molten material, without moulds, additives, or post-processing requirements. The implants are characterized by a tailored distribution of low-resorbability multiphasic CaP at the core and a highly reactive BG at the surface, which are connected through the formation of a bioactive sodium calcium phosphate interface. The bioactivity and degradation rates of the BG precursor particles are preserved after deposition of the external layers. The versatility of this type of implant opens the door to a number of applications, such as the *in vivo* investigations to determine the optimum section and proportions of CaP and BG necessary to match the new bone ingrowth rate for each particular case.

## Experimental Section

### Synthesis of bioactive glass particles

High-purity reagents (Sigma-Aldrich) were used to synthesize bioactive glasses with compositions equivalent to those of 45S5 bioactive glass (46.1 SiO_2_, 26.9 CaO, 24.4 Na_2_O, 2.6 P_2_O_5_; molar %) and S520 bioactive glass (52.0 SiO_2_, 18.0 CaO, 20.9 Na_2_O, 2.0 P_2_O_5_, 7.1 K_2_O; molar %). The precursor mixtures were subjected to melting (1.5 h at 1400 °C) and subsequent refining for 1 h in a Pt crucible. The glass melts were rapidly quenched in de-ionized water and subsequently dried. The obtained glass frits were ground and sieved between 100 μm and 250 μm in order to improve flowability through the gas–solid phase injector.

### Synthesis of multiphasic calcium phosphate core

Crystalline calcium hydroxyapatite particles (Captal 90 Plasma-Biotal) were injected by means of an Ar (Argon-50 Praxair) conveying stream and a gas–solid injector coupled to a sealed hopper. The employed disposable substrates were preheated to 200 °C during processing. An additional Ar gas stream was injected surrounding the interaction zone to avoid substrate oxidation during the initial steps of the process. At the point where the particles impinge on the substrate, a near-infrared laser beam (wavelength λ = 1064 nm, high-power Nd:YAG laser, Rofin) was focused by means of an aberration-corrected doublet lens (focal length: 80 mm). The calcium phosphate part was created by controlling the three-dimensional relative movement between the working head and the substrate. The HA mass flow rate was kept constant at 15 mg/s with a volumetric flow of carrying gas equal to 2.0 L/min and protective inert gas of 10 L/min. The processing energy density (*P*/*vD*, where *P* is the laser optical power, *v* is the scanning speed, and *D* is the laser beam diameter) was varied between 120 and 150 J/mm^2^. The relative temperature of the surface during cooling was recorded by means of a pyrometer; the cooling speed was maintained at approximately 100 °C/min. The profile of the processed part after depositing each layer was monitored by means of a CCD camera in order to maintain the geometrical configuration between the laser beam, the powder stream, and the processed part.

### Coating with bioactive glass

After depositing the last superimposed strip, the calcium phosphate core is rotated to the required position for coating with the bioactive glass. The bioactive glass particles are injected in the interaction zone by means of a second injector. In this step of the process, the successively deposited bioactive glass strips are not superimposed, but overlapped to produce a bi-dimensional coating. The laser beam and the powder stream is defocused in this second configuration to cover a higher surface area; the bioactive glass mass flow was kept constant at 20 mg/s. A value of 45 J/mm^2^ was selected for the processing energy density of the near-infrared laser beam (wavelength λ = 1064 nm) for bioactive glass thicknesses under 500 μm. For thicknesses above 500 μm, a longer wavelength λ = 10600 nm (high-power CO_2_ laser, Rofin) was employed to increase the energy coupling to the silicate glass network; this laser source was operated at an energy density of 25 J/mm^2^. The volumetric flow of carrying gas was equal to 1.0 L/min and a reduced protective gas flow of 2.0 L/min was employed.

### Immersion test in Tris-buffer

Processed samples were placed in separate plastic containers with 150 mL of 0.05 M Tris-HCl buffer (Tris(hydroxyl)methyl-aminomethane-HCl) of pH 7.4 at 36.5 °C (standard ISO10993-14). A sample surface area-to-buffer solution ratio of 0.015 cm^2^/mL was employed. Immersed samples and the solution were kept at 36.5 °C under stirring. At each of the following time periods, one 6-mL aliquot of solution was taken: 0.5, 1, 3, 6, 10, 24, 48, 72, 168, 336, and 504 h. Control samples of only 0.05 M Tris-HCl buffer were used to discard any influence of the test process in the final results. In addition, precursor 45S5 bioactive glass cast samples with the same dimensions as the laser-processed samples were tested for comparison purposes. Ten replicas of each different material were tested. Each solution was filtered through sterile filters to remove solids from the liquid and centrifuged to ensure separation of any residual solid. Spectroscopic elemental analyses were performed with inductively coupled plasma–optical emission spectroscopy (ICP-OES, Perkin Elmer Optima 4300 DV). Calibration for Ca, Si, P, Na, and K analyses were performed with 10-mg/L standards with RSD values below 2.0%.

### Cell culture

The biological performance of the material was assessed by using the pre-osteoblastic cell line MC3T3-E1. This cell line has been established from C57BL/6 mouse calvaria, and was obtained from the European Collection of Cell Cultures (ECACC, UK). The cells were cultured in MEM-alpha (Sigma, USA) supplemented with 10% fetal bovine serum (FBS; Invitrogen, USA) and were maintained at 37 °C in a humidified atmosphere with 5% CO_2_. Laser-cladding-processed samples with an area of 10 × 10 mm^2^ were cleaned in an ultrasound bath with ethanol and acetone, followed by air drying inside a laminar flow chamber. Subsequently, the samples were autoclaved for 15 min at 121 °C.

### *In vitro* cytotoxicity

The cytotoxicity of the material was determined in a non-direct-contact cells-material assay by means of the solvent extraction test, according to the EN ISO 10993-5 and 10993-12 standards. This test consists of cell seeding over the solvent extracts obtained previously. The solvent extraction was carried out by placing the sterilized processed samples on a rotating mixer for 24 h in MEM-alpha supplemented with FBS at 37 °C. The extracts were diluted with MEM-alpha to obtain 100%, 50%, 30%, 10%, and 5% of the original concentration. The same procedure was followed with phenol solution at a concentration of 6.4 g/L in MEM-alpha as the positive control. Pure MEM-alpha was used as negative control. MC3T3-E1 cells were cultured for 24 h in 96-well tissue culture plates at a concentration of 1.7 × 10^5^ cells/mL. Cellular activity was quantified with the Cell Proliferation Kit I (MTT) from Roche Molecular Biochemicals. This colorimetric assay is based on the reduction of the yellow tetrazolium salt MTT (3-(4,5-dimethyltyazolyl-2)-2,5-diphenyl tetrazolium bromide) into insoluble purple formazan crystals by the mitochondrial enzyme succinate dehydrogenase, only present in living cells. Data analysis was performed using one-way analysis of variance, Tukey post hoc test (SPSS), *p*-values < 0.05 were considered statistically significant. Data are presented as mean ± standard deviation (n = 10). Error bars in figures represent standard deviations.”

### Cell morphology and adhesion

Cell adhesion and morphology was assessed in a direct-contact cells-material assay. Sterilized samples were placed in 24-well tissue cultured plates. An MC3T3-E1 suspension of 2.5 × 10^4^ cells/mL in 1 mL of MEM-alpha supplemented with 10% FBS was added to each well, and the culture medium was renewed every 2–3 days. Cell seeding was carried out directly over the modified surface, which was the section analyzed. After each incubation time (1, 3, and 7 days), ten replicates of each experiment were prepared for analysis by scanning electron microscopy: In brief, the surfaces were fixed with 2% glutaraldehyde in 0.1 M cacodylate buffer at pH 7.4 for 2 h at 4 °C. Samples were then washed three times for 30 min each with 0.1 M cacodylate buffer and dehydrated in graded ethanol solutions (30%, 50%, 70%, 80%, and 95%) for 30 min in each solution and in absolute ethanol for 1 h. After the dehydration, samples were subjected to an increasing amylacetate/ethanol mixture (25:75, 50:50, and 75:25 for 15 min each) and to pure amylacetate twice for 15 min. The critical point of CO_2_, at 75 atm and 31.3 °C was the final step. The samples were finally mounted on metal stubs and sputter-coated with gold prior to observation by scanning electron microscopy.

### Mechanical properties

The compressive strength was determined by crushing eight CaP-BG cylindrical samples (diameter 8 mm) in dry conditions using an axial testing machine equipped with a 50 kN load cell and applying a ramp rate of 0.1 mm/min. The Vickers microhardness HV0.5 was measured by a microhardness testing machine, applying a load of 4.903 N during a period of 15 s to the bioactive glass surface and to the CaP core cross-section, respectively. The fracture toughness *K*_IC_ was evaluated, just after indentation, from the well-developed radial cracks^58^. A value of 95 GPa was employed for the Young’s modulus for the CaP inner core and 78 GPa for the bioactive glass outer layer.

## Additional Information

**How to cite this article**: Comesaña, R. et al. Toward Smart Implant Synthesis: Bonding Bioceramics of Different Resorbability to Match Bone Growth Rates. *Sci. Rep.* **5**, 10677; doi: 10.1038/srep10677 (2015).

## Supplementary Material

Supporting Information

## Figures and Tables

**Figure 1:**
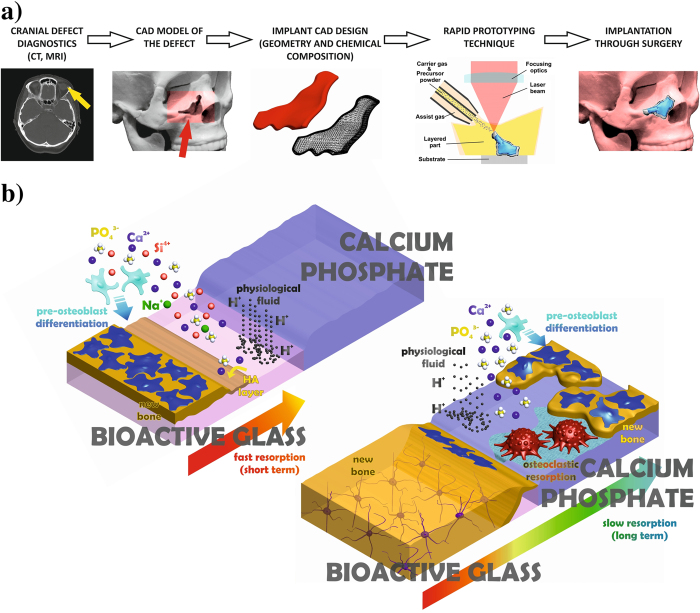
Schematic illustration of implant tailored fabrication from defect diagnostics, and of the approach followed in this work to attain a progressively resorbed material. (**a**) Computerized imaging techniques provide three-dimensional data of the bone defect, which are employed to design a computer model of the required implant, afterward fabricated by a rapid prototyping technique. (**b**) The outer bioactive glass (BG) layer (upper left) of the implant is resorbed within an initial stage by the physiological fluid, favoring the precipitation of an osteoconductive HA layer and the differentiation of osteoblast precursors required for intensive bone formation. Subsequently, the inner multiphasic CaP is slowly resorbed by mediation of osteoclasts and physiological fluid, releasing chemical species that promote bone ingrowth and providing stability at long term.
