# Supplementary material for: Toward Smart Implant Synthesis: Bonding Bioceramics of Different Resorbability to Match Bone Growth Rates
Source: Sci Rep. 2015 Jun 2;5:10677. doi: 10.1038/srep10677 (PMC4451530; doi:10.1038/srep10677)
Supplement: Supporting Information [file srep10677-s1.doc]

**Supporting Information**

**Toward smart implant synthesis: Bonding bioceramics of different resorbability to match bone growth rates**

*Rafael Comesaña, Fernando Lusquiños, Jesús del Val, Félix Quintero, Antonio Riveiro, Mohamed Boutinguiza, Julian R. Jones, Robert G. Hill, Juan Pou**

Prof. J. Pou

Applied Physics Dpt., University of Vigo,

E.I.I., Lagoas-Marcosende

E-36310, Vigo, Spain

jpou@uvigo.es

**FTIR and Raman spectroscopy spectra analysis of CaP-BG samples cross-section.**

The averaged FTIR spectra of precursor materials and from different zones in across the CaP‑BG samples cross-section are shown in Figure S1. The bands and peaks observed in the spectra are listed in Table S1. In the spectra acquired from the 45S5 BG zone, broad bands at 923 and 1047 cm^-1^ are present, which are related to the Si–O–NBO stretching vibration mode (NBO: non-bridging oxygen) and Si–O–Si stretching vibration mode, respectively. In addition, a weak shoulder is observed at 860 cm^-1^ and attributed to the Si–O–2NBO stretching mode. Similarly, the processed S520 BG zone exhibits broad Si–O–NBO and Si–O–Si stretching bands at 925 and 1052 cm^-1^, respectively. Due to the lower quantity of Q^2^ units (Q*^n^* unit: tetrahedral unit formed by one Si central atom and *n* bridging oxygens) present in both reference S520 BG and the BG deposited layer, no shoulders are observed corresponding to Si–O–2NBO groups. Regarding the interface zone, in the CaP-45S5 samples, the phosphate group symmetric stretching mode (ν_1_ PO_4_^3-^) is present as a weak peak at 961 cm^‑1^, together with the antisymmetric stretching modes (ν_3_ PO_4_^3-^) as strong peaks (1060, 1083, and 1093 cm^‑1^) and shoulders between 1024 and 1120 cm^-1^. Similar spectra are observed for the CaP–BG S520 interface, although the shoulder positions are less marked in the ν_3_ PO_4_^3-^ band. The spectra acquired from the CaP core in both sample types shows a very weak ν_1_ PO_4_^3-^ shoulder at 960 cm^-1^ and marked splitting of the ν_3_ PO_4_^3-^ modes strongly between 991 and 1114 cm^‑1^ due to decomposition of the HA precursor material in α‑TCP and TTCP^S1^.

| **CaP-45S5BG** |  | **CaP-S520BG** |
| --- | --- | --- |
| **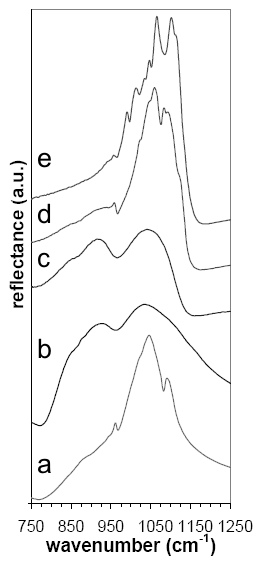** |  | 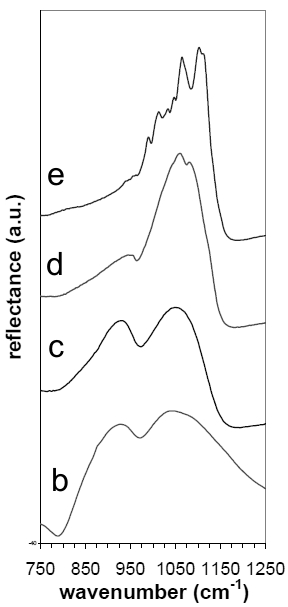 |

**Figure S1.** FTIR spectra of (a) calcium hydroxyapatite precursor material, (b) BG precursor material, and from cross sections taken from different zones in the CaP-BG samples: (c) BG layer close to interface, (d) interface zone, and (e) CaP core close to interface.

**Table S1. FTIR modes between 750 and 1250 cm^-1^ and assignments at room temperature (±2 cm^-1^).^S1,S2^**

|  |  |  |  |  |  |  |  |  |
| --- | --- | --- | --- | --- | --- | --- | --- | --- |
|  |  |  |  |  |  |  |  |  |
| HA Captal90 | BG-CaP CaP core | 45S5-CaP interface | S520-CaP interface | 45S5-CaP BG layer | 45S5 BG (reference) | S520-CaP BG layer | S520 BG  (reference) | Assignment |
|  |  |  |  |  |  |  |  |  |
|  |  |  |  | 860 sh | 860 sh |  |  | Si-O-2NBO |
|  |  |  |  |  |  |  |  |  |
|  |  |  |  | 923 br | 931br | 934 br | 933 br | Si-O-NBO |
|  |  |  |  |  |  |  |  |  |
| 961 w | 960 w | 961 w | 955 w |  |  |  |  | ν1 PO4 |
|  |  |  |  |  |  |  |  |  |
|  |  |  |  | 1047 br | 1041 br | 1054 br | 1051 br | Si-O-Si (s) |
|  |  |  |  |  |  |  |  |  |
|  | 991 m |  |  |  |  |  |  | ν_3_ PO_4_ |
|  | 1014 m | 1024 sh |  |  |  |  |  |  |
| 1047 br | 1049 m | 1045 sh |  |  |  |  |  |  |
| 1091 m | 1066 s | 1060 s | 1065 s |  |  |  |  |  |
|  | 1105 s | 1083 m | 1084 s |  |  |  |  |  |
|  | 1114 m | 1093 m |  |  |  |  |  |  |
|  |  | 1120 sh |  |  |  |  |  |  |
|  |  |  |  |  |  |  |  |  |

(s:strong; m: medium; w: weak; sh: shoulder; br: broad band)

**Table S2. Raman modes and assignments at room temperature (±1 cm^-1^).^S2,S3^**

|  |  |  |  |  |  |  |  |  |
| --- | --- | --- | --- | --- | --- | --- | --- | --- |
|  |  |  |  |  |  |  |  |  |
| HA Captal90 | BG-CaP CaP core | 45S5-CaP interface | S520-CaP interface | 45S5-CaP BG zone | 45S5 BG (reference) | S520-CaP BG zone | S520 BG (reference) | Assignment |
|  |  |  |  |  |  |  |  |  |
|  |  | 218 w | 214 w |  |  |  |  | Ca-PO_4_ lattice |
|  |  | 247 w | 244 w |  |  |  |  |  |
|  |  |  |  |  |  |  |  |  |
| 284 w |  |  |  |  |  |  |  |  |
|  |  |  |  |  |  |  |  |  |
|  |  |  |  | 440 w | 440 w | 438 w | 436 w | Si-O-Si (stret.) >5 Q^4^ |
|  |  |  |  |  |  |  |  |  |
|  | 391 w |  |  |  |  |  |  | ν_2_ PO_4_ |
|  | 409 m |  |  |  |  |  |  |  |
| 431 m | 420 m | 428 m | 426 m |  |  |  |  |  |
| 444 sh | 448 m | 450 m | 449 sh |  |  |  |  |  |
|  |  |  |  |  |  |  |  |  |
|  | 556 w |  |  |  |  |  |  | ν_4_ PO_4_ |
|  | 567 m |  |  |  |  |  |  |  |
| 581 m | 583 w | 567 sh | 564 sh |  |  |  |  |  |
| 591 m | 597 m | 586 m | 581 m |  |  |  |  |  |
| 607 m | 607 sh | 598 sh |  |  |  |  |  |  |
|  | 616 sh |  |  |  |  |  |  |  |
|  |  |  |  |  |  |  |  |  |
|  |  |  |  | 622 m | 621 m | 607 m | 604 m | Si-O-Si (rock) 3Q4 |
|  |  |  |  |  |  |  |  |  |
|  |  |  |  | 863 w | 863 w | 863 w | 864 w | Si-O-2NBO (stret.) |
|  |  |  |  |  |  |  |  |  |
|  |  |  |  | 948 m | 946 m | 946 m | 944 m | Si-O-NBO (stret.) |
|  |  |  |  |  |  |  |  |  |
|  | 941 sh |  |  |  |  |  |  | ν_1_ PO_4_ |
|  | 948 s |  |  |  |  |  |  |  |
|  | 958 sh |  |  |  |  |  |  |  |
| 963 s | 963 s | 966 m | 964 m |  |  |  |  |  |
|  |  |  |  |  |  |  |  |  |
|  |  |  |  | 1032 br | 1028 br | 1074 br | 1075 m | Si-O-Si (stret.) |
|  |  |  |  |  |  |  |  |  |
|  | 1008 w | 1013 m | 1012 m |  |  |  |  | ν_3_ PO_4_ |
| 1030 sh | 1027 w | 1024 m | 1023 sh |  |  |  |  |  |
| 1049 m | 1050 w | 1046 m | 1043 m |  |  |  |  |  |
| 1078 m | 1078 w |  |  |  |  |  |  |  |
|  | 1098 w |  |  |  |  |  |  |  |
|  | 1122 w |  |  |  |  |  |  |  |
|  | 1133 w |  |  |  |  |  |  |  |
|  |  |  |  |  |  |  |  |  |

(s:strong; m: medium; w: weak; sh: shoulder; br: broad band)

**Network connectivity analysis of the BG external layers.**

The observed diffusion of Na^+^ from the BG coatings to the interface, and of Ca^2+^ cations from the interface to the BG coating (Figure 4b in main text), induces compositional change of the BG coating in proximity of the interface. The reaction can be expressed as follows:

Ca_3_(PO_4_)_2_ + 2 Na^+^ → 2 NaCaPO_4_ + Ca^2+^ (S1)

Ca_4_(PO_4_)_2_O + 2 Na^+^ → 2 NaCaPO_4_ + 2Ca^2+^ + ½ O_2_ ↑ (S2)

Na^+^ and Ca^2+^ both act as modifier elements in the BG silica network and, therefore, network connectivity variation is minimized by substitution of Na^+^ by Ca^2+^ (glass network connectivity NC = 4 – *NBO/T*, where *NBO/T* is the number of non-bridging oxygens per silica tetrahedron)^S4^. Phosphorous diffusion would also play a role, increasing the network connectivity, but the observed phosphorous diffusion in the processed samples was very limited. Assuming that released Ca^2+^ cations are completely diffused into the BG, it should be noted that Equation (S1) would maintain the network connectivity as two Na^+^ cations are required to release one Ca^2+^ cation, while Equation (S2) would decrease network connectivity by liberating one Ca^2+^ cation per Na^+^ cation. Divalent cation mobility is considered to be much lower than monovalent cation mobility in silicate glasses, as demonstrated by the activation energies for DC conductivity in alkali and alkaline earth silicate glasses^[S5]^. Moreover, the combination of dissimilar ions usually leads to mobility reduction of such ions, referred as the mixed alkali effect^[S6]^. Nevertheless, for soda-lime-silicate glasses, a synergistic effect between Na^+^ and Ca^2+^ cations is observed. Increasing Na_2_O content at the expense of CaO causes an increase in Ca^2+^ mobility, and it has been suggested that Ca^2+^ diffuses via the empty sites created by Na^+[S7]^. The divalent cation mobility increases in the presence of Na^+^ as the difference between the cation radii decreases. Na^+^ and Ca^2+^ diffusion leading toward increasing similarity in both cations concentrations occurs at the interface; this enhanced cation diffusion with preference for Na–Ca pairing around NBOs was previously reported for soda‑lime‑silicate glasses^[S5,S7,S8]^.

**Supporting Information References**

1. [R. Comesaña](http://www.researchgate.net/researcher/56558328_R_Comesana/), [F. Lusquiños](http://www.researchgate.net/researcher/33995278_F_Lusquinos/), [J. del Val](http://www.researchgate.net/researcher/55047395_J_del_Val/), [T. Malot](http://www.researchgate.net/researcher/71901279_T_Malot/), [M. López-Álvarez](http://www.researchgate.net/researcher/31205589_M_Lopez-Alvarez/), [A. Riveiro](http://www.researchgate.net/researcher/57991198_A_Riveiro/), [F. Quintero](http://www.researchgate.net/researcher/26613927_F_Quintero/), [M. Boutinguiza](http://www.researchgate.net/researcher/34761219_M_Boutinguiza/), [P. Aubry](http://www.researchgate.net/researcher/71836957_P_Aubry/), [A. De Carlos](http://www.researchgate.net/researcher/33380211_A_De_Carlos/), [J. Pou](http://www.researchgate.net/researcher/7559848_J_Pou/), J. Eur. Ceram. Soc. 2011, 31, 29.
2. H. Aguiar, J. Serra, P. González, B. Leon, *J. Non-Cryst Solids* **2009**, 355, 475.
3. a) U. Posset, E. Löcklin, R. Thull, W. Kieferm, *J. Biomed. Mater. Res.* **1998**, 40, 640; b) G. Penel, G. Leroy, C. Rey, E. Bres*, Calcif. Tissue Int.* **1998**, 63, 475; c) P. González, J. Serra, S. Liste, S. Chiussi, B. León, M. Pérez-Amor, *J. Non-Cryst. Solids* **2003**, 320, 92.
4. I. Elgayar, A. E. Aliev, A. R. Boccaccini, R. G. Hill, *J. Non-Cryst .Solids* **2005**, 351, 173.
5. S.K. Lee, J.C. Stebbins, *J. Phys. Chem. B* **2003**, 107, 3141.
6. a) R.V. Caporali, *JACS* **1964**,47, 412; J.O. Isard, *J. Non-Cryst. Solids* **1969**, 1, 235; b) R.H. Doremus, *J Am Ceram Soc* **1974**, 57, 478.
7. C. Karlsson, E. Zanghellini, J. Swenson, B. Roling, D.T. Bowron, L. Borjeson, *Phys. Rev. B: Condens. Matter Mater. Phys.* **2005**, 72, 064206.
8. a) B. Roling, M.D. Ingram, *Solid State Ionics* **1998**, 105, 47; b) Roling, B. & Ingram, M.D. *J. Non-Cryst. Solids* **2000**, 265, 113; c) F. Natrup, H. Bracht, C. Martiny, S. Murugavel, B. Roling, B*. Phys. Chem. Chem. Phys.* **2002**, 4, 3225.
